# Health behaviors and tooth retention among older adults in China: findings from the 4th Chinese national oral health survey

**DOI:** 10.1186/s12903-022-02283-2

**Published:** 2022-07-14

**Authors:** Xiaoyan Ou, Liwei Zeng, Yixuan Zeng, Yaolin Pei, Xiujuan Zhang, Wei Wu, Shahrzad Siamdoust, Bei Wu

**Affiliations:** 1 Key Laboratory of Oral Biomedicine, Jiangxi Province, Jiangxi Province Clinical Research Center for Oral Diseases, Affiliated Stomatological Hospital of Nanchang University, 49 Fuzhou Road, Nanchang, 330006 Jiangxi Province China; 2 Rory Meyers College of Nursing, New York University, 433 First Avenue, New York, NY 10010 USA; 3 Rory Meyers College of Nursing and NYU Aging Incubator, New York University, 433 First Avenue, New York, NY 10010 USA

**Keywords:** Tooth loss, Oral health behavior, Ageing, China

## Abstract

**Background:**

This study aimed to examine the association between oral health behaviors and tooth retention among Chinese older adults.

**Methods:**

Data were used from the 4th Chinese National Oral Health Survey, a nationally representative sample. The sample included 9054 older adults aged 55 to 74. Control variables and oral health behaviors were measured through a questionnaire interview, and the number of remaining teeth and periodontal health were obtained from an oral health examination. A chi-square test was used for univariate analysis. Multivariate Logistic regression was used to explore the association between health behaviors and the number of remaining teeth.

**Results:**

The average number of remaining teeth in the sample was 24.4 ± 7.7. There was a higher proportion of older adults living in urban areas with 20 or more teeth than those living in rural areas (83.2% vs. 79.4%, *P* < 0.001); and a higher proportion of individuals with high education levels with 20 or more teeth compared to those with low education levels (*P* < 0.001). Logistic regression models showed that older adults who used toothpicks `(OR = 3.37, 95% CI 2.94–3.85), dental floss (OR = 1.93, 95% CI 1.05–3.53), toothpaste (OR = 3.89, 95% CI 3.14–4.83); and never smoked (OR = 1.43 95% CI 1.20–1.70) were more likely to retain 20 or more natural teeth; whereas older adults who had a dental visit were less likely to retain 20 or more natural teeth (OR = 0.45, 95% CI 0.39–052).

**Conclusion:**

Good oral hygiene practices, never smoking, and regular dental visits focusing on prevention are significantly associated with teeth retention. It is critical to promote a healthy lifestyle and improve prevention-oriented oral health care systems.

## Background

The World Health Organization (WHO) states that oral health is a key indicator of overall health [1]. Poor oral health among older people has been evident worldwide, such as high caries activity, high prevalence rates of periodontal disease, severe tooth loss, dry mouth, and oral precancer/cancer [2]. Tooth loss is an indicator of poor oral health. Extensive tooth loss impairs chewing efficiency [3] and has a negative impact on oral health-related quality of life (OHRQoL) [4, 5]. Studies have shown correlations between tooth loss and many chronic diseases, including hypertension, cardiovascular disease, Alzheimer's disease, diabetes, and mortality [6–11]. Preventive oral health research have been focused on controlling the occurrence of oral diseases through lifestyle, as well as the control of common risk factors for oral health and general health [12]. Therefore, there is a need to examine the association between health behaviors and tooth retention.

Currently, many scholars are focusing on the related factors of oral diseases in older adults. Few studies have examined the association between health behaviors and the number of remaining teeth. Previous intervention studies have demonstrated that the prevention and control of dental caries can contribute to the decline of tooth loss [13]. An individual’s lifestyle and behaviors play an important role in the prevention and control of tooth loss. However, most studies have been conducted in Western countries [14–18]; few studies examined the association between health behaviors and oral health among the Chinese population. There are significant differences in the individual behaviors and oral health care system between Western countries and China. For example, in China, the use of toothpicks is a common behavior, which is different from the West [19]. Chinese oral health care system still focuses on the treatment rather than prevention. Therefore, the findings on the association between health behaviors and tooth loss from existing literature cannot be generalizable to Chinese settings without further research.

Data from the 4th Chinese National Oral Health Survey showed that oral health problems were common in Chinese adults: only 13.8% had complete dentition, 84.4% had dentition defects, and 1.8% were edentulous [20]. Severe tooth loss causes high disease burden on the population. One study found that oral diseases cause 15 million disability adjusted life-years (DALYs) globally. For severe periodontitis, untreated tooth decay and severe tooth loss, the disability weights are 0.0079, 0.012, and 0.073, respectively [21]. The purpose of this study was to examine the association between oral health behaviors and teeth retention among Chinese older adults. The findings of this study will provide knowledge and insight to develop oral health policies and interventions aimed at improving tooth retention among the Chinese adult population.

## Methods

### Sampling method

Cross-sectional data from the 4th National Oral Health Survey of China conducted in 2015–2016 were used in this study, we used data that were collected by the Chinese Centre for Disease Control and Prevention [22]. The survey included oral health status and health behaviors of participants. A multi-stage cluster sampling method was adopted for the identification of participants. All 31 provinces, autonomous regions and municipalities of Mainland China were included. First, 2 urban and 2 rural districts (defined by the National Bureau of Statistics of China) were selected in each province using a probability proportional to size (PPS) sampling. The local Centre for Disease Control and Prevention of each province was responsible for the identification of the districts. In total, 62 urban and 62 rural districts were selected. 3 sub-districts (referred to as “streets” in urban districts and as “townships” in rural districts) were then selected using the PPS sampling method in each district. In brief, a total of 186 streets and 186 townships were selected. The following age groups: 35 to 44; 55 to 64; and 65 to 74 years, were consecutively recruited. There were 36 local residents with a male to female ratio of 1:1 [23]. A total of 13,464 people participated in the survey. After removing individuals under age 55, the study sample had 9054 older adults aged 55 to 74.

### Oral health examination

Each participant was asked to undergo a professional oral examination. The examiners were dental professionals who had engaged in oral clinical work for more than 3 years and were qualified as dental practitioners; and the recorders were clinicians and/or nurses with relevant clinical experience. A disposable dental mirror, an intraoral light-emitting diode light, and a ball-ended WHO Community Periodontal Index (CPI) probe were used in the examination. The diagnostic criteria were followed using the WHO recommendations [24]. Prior to the launch of the on-site data collection, calibration training programmes were conducted to ensure the reliability of the results. The standard reliability training was conducted for each examiner, in which the kappa value of caries status was greater than 0.8, and the Kappa value of periodontal pocket depth was above 0.6. In order to monitor the inter-examiner reliability of the data collection, 5% of the participants were randomly selected for a second examination at the same day of the data collection. “Periodontally healthy” was defined as: 1 = no bleeding, no periodontal pockets, and no loss of attachment greater than 3 mm of all remaining teeth; and 0 = bleeding, periodontal pockets, and loss of attachment greater than 3 mm of all remaining teeth.

### Measures

Dependent variable: Many studies have shown that to obtain satisfactory function and aesthetics, at least 20 natural teeth are required [5, 23, 25], so participants were categorized as those with at least 20 or more natural teeth and those with less than 20 natural teeth. Loss of 8 teeth (excluding wisdom teeth) is commonly considered as significant tooth loss [26]. Those with mobility or noted to be extracted was considered as a remaining tooth. The number of remaining teeth was coded as (0 = number of remaining teeth < 20, 1 = number of remaining teeth ≥ 20).

In-person structured questionnaire interviews were conducted by trained interviewers. We included the following measures:

The demographic variables included sex (1 = female), age (1 = 65–74), residence (1 = living in rural areas), educational level, occupation and region. We coded educational levels by years of education: 1 =  ≤ 6 years of education, 2 = 7–9 years of education, 3 = 10–12 years of education, and 4 =  ≥ 13 years of education. Occupation was coded as (0 = Non-white collar, 1 = heads of state organs, party and mass organizations, enterprises and institutions, professional and technical personnel, and military personnel). We used two dummy variables to present region with the eastern area as the reference group: 1 = western area, and 2 = central area.

We also controlled for self-reported oral health and general health. Self-reported oral health was coded as: 0 = fair/poor, 1 = good. Self-reported general health was coded as: 0 = fair/poor, 1 = good.

Health behaviors included frequency of tooth brushing, use of toothpicks (0 = never, 1 = ever), dental floss (0 = never, 1 = ever), toothpaste (0 = never, 1 = ever), and fluoride toothpaste (0 = never, 1 = ever), smoking (0 = current smoker, 1 = never, 2 = ex-smokers), alcohol drinking (0 = ever, 1 = never), dental visit (0 = no, 1 = yes), and tooth scaling (0 = never, 1 = ever). We used two dummy variables to present toothbrushing frequency with less than one time per day as the reference group: one time per day, and two times or more times per day, respectively. Dental visit was collected by asking the participant if they have dental visits within the last 12 months: 0 = no, 1 = yes.

### Statistical analysis

The description of measurement data was expressed as mean ± standard error (SE). A chi-square test was used for univariate analysis of general characteristics, health behaviors, self-reported oral health, self-reported general health, and number of remaining teeth. Multivariate logistic regression was used to explore the association between health behaviors and the number of remaining teeth. The odds ratios (OR), 95% confidence intervals (CI), and the *p*-value were used to indicate the degree of association between the independent variable and the dependent variable. This study established three models to explore the association between health behaviors and the number of remaining teeth. Model 1 was Univariate logistic regression, Model 2 was Multivariate logistic regression analysis without control variables, and Model 3 was Multivariate logistic regression analysis after adjusting age, sex, residence, region, education level, occupation, periodontal health, self-reported dental health, and self-reported general health. The statistical analysis was performed using IBM SPSS statistics 19.0 software, using alpha level of 0.05.

## Results

Table 1 shows participants’ characteristics by the number of remaining teeth. The average age was 64.4. The average number of remaining teeth in 9054 cases was 24.4 ± 7.7. The percentage of participants with more than 20 teeth was 81.3%. There were significantly more urban residents (51.8%) with 20 or more teeth than rural residents (48.2%) (*P* < 0.001). The higher the level of education, the higher the proportion of participants with 20 teeth or more (all *P*-values and p for trends < 0.001).

**Table 1:** General characteristics of a sample of Chinese population (n = 9054) according to number of remained teeth

Variable	Category	No. of population	Ratio	Number of retention teeth < 20		Number of retention teeth ≥ 20		*χ*^2^	*P*-Value
				n	%	n	%		
Age(yr)	55–64	4623	51.1	503	29.7	4120	56.0	379.840	< 0.001
	65–74	4431	48.9	1190	70.3	3241	44.0		
Sex	Male	4514	49.9	865	51.1	3649	49.6	1.273	0.135
	Female	4540	50.1	828	48.9	3712	50.4		
Residence	Urban	4587	50.7	773	45.7	3814	51.8	20.862	< 0.001
	Rural	4467	49.3	920	54.3	3547	48.2		
Region	Eastern	3196	35.3	598	35.3	2598	35.3	8.562	0.014
	Western	3565	39.4	709	41.9	2856	38.8		
	Central	2293	25.3	386	22.8	1907	25.9		
Education level (yrs)	≤ 6	4803	53.1	1116	65.9	3687	50.1	148.168	< 0.001
	7–9	2370	26.2	359	21.2	2011	27.3		
	10–12	1387	15.3	168	9.9	1219	16.6		
	13 ≤	494	5.5	50	3.0	444	6.0		
Occupation	Non-white collar	8490	93.8	1605	94.8	6885	93.5	3.792	0.027
	White collar	564	6.2	88	5.2	476	6.5		
Periodontal health	No	8411	92.9	1330	78.6	7081	96.2	649.000	< 0.001
	Yes	643	7.1	363	21.4	280	3.8		
Self-reported oral health	Fair/poor	6734	74.4	1450	85.6	5284	71.8	138.801	< 0.001
	Good	2320	25.6	243	14.4	2077	28.2		
Self-reported general health	Fair/poor	5485	60.6	1067	63.0	4418	60.0	5.205	0.012
	Good	3569	39.4	626	37.0	2943	40.0		
Total		9054	100.0	1693	100.0	7361	100.0		

**Fig. 1:**
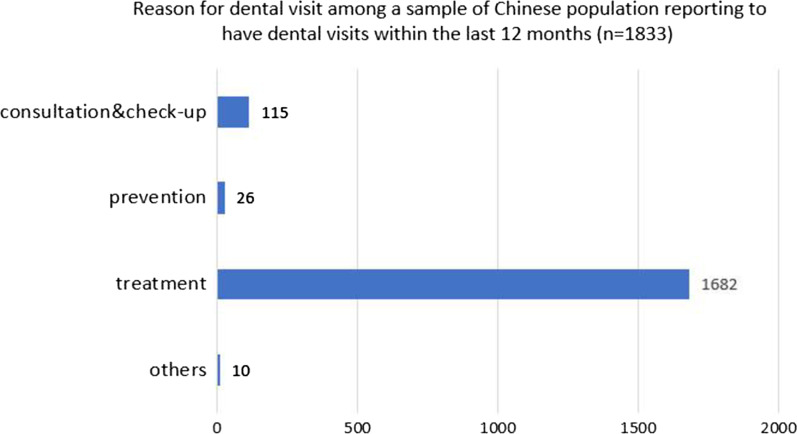
Reason for dental visit among a sample of Chinese population reporting to have dental visits within the last 12 months (n = 1833)

Table 2 presents the comparison results in health behaviors between participants who had 20 or more teeth and those who had less than 20 teeth. Participants had significant differences in the frequency of brushing, whether or not they used toothpicks, dental floss, toothpaste, fluoride toothpaste, smoking, drinking, and dental visits. Figure 1 shows the reasons for dental visits. Treatment rather than prevention was the major reason for dental visits; 92% reported the reason for their last dental visit was for treatment.

**Table 2:** Health behaviors of a sample of Chinese population (n = 9054) according to number of remained teeth

Variable	Category	No. of population	Ratio	Number of retention teeth < 20		Number of retention teeth ≥ 20		*χ*^2^	*P*-Value
				n	%	n	%		
Toothbrushing frequency	< 1/day	1503	16.6	473	27.9	1030	14.0	193.501	< 0.001
	1/day	4802	53.0	782	46.2	4020	54.6		
	≧2/day	2749	30.4	438	25.9	2311	31.4		
Using tooth picks	Never	5029	55.5	1338	79.0	3691	50.1	465.202	< 0.001
	Ever	4025	44.5	355	21.0	3670	49.9		
Frequency of using dental floss	Never	8874	98.0	1680	99.2	7194	97.7	15.912	< 0.001
	Ever	180	2.0	13	0.8	167	2.3		
Using toothpaste	Never	704	7.8	352	20.8	352	4.8	491.963	< 0.001
	Ever	8350	92.2	1341	79.2	7009	95.2		
Fluoride toothpaste	Never	8063	89.1	1574	93.0	6489	88.2	32.769	< 0.001
	Ever	991	11.0	119	7.0	872	11.8		
Smoking	Current smokers	2316	25.6	481	28.4	1835	24.9	13.629	0.001
	Never	5606	61.9	982	58.0	4624	62.8		
	Ex-smokers	1132	12.5	230	13.6	902	12.3		
Drinking alcohol	Ever	2983	33.0	483	28.5	2500	34.0	18.394	< 0.001
	Never	6071	67.1	1210	71.5	4861	66.0		
Dental visit	Never	3008	33.2	394	23.3	2614	35.5	92.938	< 0.001
	Ever	6046	66.8	1299	76.7	4747	64.5		
Tooth scaling	Never	8792	97.1	1653	97.6	7139	97.0	2.090	0.171
	Ever	262	2.9	40	2.4	222	3.0		
Total		9054	100.0	1693	100.0	7361	100.0

Table 3 shows the multivariate analysis of the association between oral health behaviors and number of teeth retained. In Model 1, univariate logistic regression shows that all of the health behaviors were associated with the number of remaining teeth without controlling for any other variables. When adding all of the health behaviors in Model 2, each health behavior was still significantly associated with the number of remaining teeth. However, when adding age, sex, residence, region, education level, occupation, periodontal health, self-reported dental health, and self-reported general health in Model 3, the frequency of toothbrushing, the use of fluoride toothpaste, drinking and tooth scaling were no longer significantly associated with the number of remaining teeth. We only found that the use of toothpicks (OR = 3.37, 95% CI 2.94–3.85), dental floss (OR = 1.93, 95% CI 1.05–3.53), toothpaste (OR = 3.89, 95% CI 3.14–4.83), smoking (OR = 1.43, 95% CI 1.20–1.70), and dental visits (OR = 0.45, 95% CI 0.39–0.52) were significantly associated with the number of remaining teeth.

**Table 3:** Multivariate analysis between health behavior and the number of remaining teeth among a sample of Chinese population (n = 9054)

Variable	Category	Number of retention teeth ≥ 20(%)	Model1		Model2		MODEL3	
			OR	95%CI	OR	95%CI	OR	95%CI
Toothbrushing frequency	< 1/day	14.0	1		1		1	
	1/day	54.6	5.14^***^	4.76–5.55	1.30^**^	1.09–1.54	1.17	0.98–1.41
	≧2/day	31.4	5.28^***^	4.76–5.84	1.33^**^	1.1–1.60	1.21	0.98–1.49
Using tooth picks	Never	50.1	1		1		1	
	Ever	49.9	10.34^***^	9.27–11.53	3.56^***^	3.13–4.05	3.37^***^	2.94–3.85
Using dental floss	Never	97.7	1		1		1	
	Ever	2.3	12.85^***^	7.31–22.59	2.36^**^	1.31–4.24	1.93^*^	1.05–3.53
Using toothpaste	Never	4.8	1		1		1	
	Ever	95.2	5.23^**^	4.93–5.54	4.78^***^	3.95–5.78	3.89^***^	3.14–4.83
Fluoride toothpaste	Never	88.2	1		1		1	
	Ever	11.8	7.33^***^	6.05–8.88	1.42^**^	1.15–1.75	1.18	0.94–1.47
Smoking	Current Smokers	24.9	1		1		1	
	Never	62.8	4.71^***^	4.40–5.04	1.50^***^	1.31–1.73	1.43^***^	1.20–1.70
	Ex-smokers	12.3	3.92^***^	3.39–4.53	1.11	0.92–1.34	1.16	0.95–1.42
Drinking alcohol	Ever	34.0	1		1		1	
	Never	66.0	2.26^***^	2.19–2.33	0.85^***^	0.77–0.93	0.93	0.81–1.06
Dental visit	Never	35.5	1		1		1	
	Ever	64.5	3.65^***^	3.44–3.89	0.42^***^	0.37–0.48	0.45^***^	0.39–0.52
Tooth scaling	Never	97.0	1		1		1	
	Ever	3.0	5.55^***^	3.96–7.77	1.25	0.87–1.79	1.10	0.75–1.61

^*^P < 0.05, **P < 0.01, ***P < 0.001

Model1: Univariate logistic regression

Model2: Multivariate logistic regression analysis,

Model3: Multivariate logistic regression analysis, Adjusted age, sex, residence, region, education level, occupation, periodontal health, Self-reported dental health, Self-reported general health

## Discussion

By using the 4th Chinese National Oral Health Survey, the study aimed to investigate the association between tooth retention and health behaviors among Chinese older adults. We found that participants who had healthy behaviors were more likely to have more than 20 teeth compared to those without healthy behaviors. Moreover, we found that the use of toothpicks, dental floss, toothpaste, smoking, and dental visits were significantly associated with the number of remaining teeth. The frequency of brushing was not significantly related to the number of remaining teeth in older adults, which was different from previous studies that showed higher frequency of toothbrushing was associated with more remaining teeth [27]. This may be because respondents did not brush their teeth properly and/or the frequency of toothbrushing was low [28], therefore, their oral health status was not ideal. Some Chinese scholars have found that Chinese adults' attention to oral health is behind developed countries and good oral hygiene behaviors were not formed [29]. This may explain why the frequency of toothbrushing was not associated with the higher number of remaining teeth in China. This suggests that when we want to enhance people’s awareness of oral health, especially for those aged 55 or older, proper brushing techniques should be signaled. The use of toothpicks, dental floss, and toothpaste is conducive to the retention of teeth. As toothpicks and dental floss are used to get rid of food debris between teeth, toothpaste as a cleaning agent can better help clean teeth and remove plaque and slow down the accumulation of dental calculus. Good oral hygiene can effectively keep or improve periodontal health, as periodontitis is mainly accountable for tooth loss in adults.

Older adults who never smoked were more likely to have more than 20 remaining teeth than those who were current smokers. Tobacco contains a variety of harmful substances that can directly stimulate periodontal tissues or enter the bloodstream, causing periodontal damage. Azodo's research on Nigerian residents' smoking and oral health behaviors found that smokers had poorer subjectively rated periodontal health and poorer oral self-care behavior [30], which is similar to our study.

People who had more dental visits were less likely to have more than 20 remaining teeth. This may be because Chinese adults seek dental services for treatment rather than prevention and patients who seek dental consultations have poor oral conditions [31], which is different from what is seen in studies from developed countries [32]. Secondly, Chinese scholars have found that 28% of Chinese older adults have no remaining teeth and that only 19% had dental care in the past year [33]. This demonstrates that people with dental problems are not active in seeking dental care. The third reason is that the treatment is not performed at the optimal time, resulting in pain and lack of other treatment options as the main reasons for tooth extraction [34]. Murakami et al. and other scholars reported that once oral diseases occur, due to the lack of dental insurance and costly treatments, older adults are reluctant to seek timely dental treatment [35]. We found that only 20.2% of the 9,054 participants reported having dental visits, and 92% of those who had dental visits reported that their last dental visits were to seek treatment. Therefore, oral health interventions and programs should actively promote regular dental visits with the long-term goal of maintaining oral health in China.

The average life expectancy of a Chinese resident is increasing every year. According to the data released by National Health Commission of the People’s Republic of China [36], the average life expectancy of a Chinese resident in 2018 was 77.0 years, which means that the length of life among older adults whether they are 60 or 80 years old will be extended. Multi-dimensional improvement of the quality of life of older adults has become a research priority [2]. Oral health is an important part of general health, and it is essential to provide oral health care throughout the life course. Because oral diseases, such as missing teeth, are inextricably linked to the occurrence of other diseases [37]. Oral health is often neglected, especially in developing countries. Findings from our study suggest that it is important to develop an oral health care system with a focus on prevention and promote healthy behaviors in these developing countries. Based on this, it is necessary to start with oral hygiene behaviors to improve the life of natural teeth and maintain oral function, therefore improving the overall health and quality of life of older adults. Health administrative departments should continue to promote the construction of medical associations when implementing the ‘Healthy oral action plan’ (2019–2025) [38]; formulate regional oral health development plans for older adults in different regions, especially for those from rural areas; and provide quantitative oral health funding subsidies to low-income populations to improve access to health care and promote the transformations of disease treatments in health management [39]. In order to help older adults have better oral health behaviors and retain natural teeth, local communities need to develop more oral health education programs. Findings from our study suggest that it is important to develop an oral health care system with a focus on prevention and promotion of healthy behaviors. Oral health professionals need to work with other professionals collaboratively to achieve optimal oral health outcomes for this age group globally.

There are several limitations in the study. First, due to the cross-sectional nature of the study, we were not able to examine a causal relationship between health behaviors and the number of remaining teeth. Second, there may have been bias in the oral hygiene behavior due to the self-reported nature of the study. However, the present study also has strengths. We used a representative sample of the whole Chinese population using the 4th Chinese National Oral Health Survey. This oral health survey is the only large national study that includes clinical examinations of oral health in older adults. Also, we controlled for both the subjective and objective oral health statuses in this study.

In conclusion, good oral hygiene practices, never smoking, and regular dental visits focusing on prevention are significantly associated with teeth retention. It is critical to promote a healthy lifestyle and improve prevention-oriented oral health care systems. Future studies are needed to develop interventions to help people retain more teeth.

## Data Availability

The datasets used and/or analyzed during the current study are available from the corresponding author on reasonable request for all interested researchers.

## Abbreviations

WHO: The world health organization
OHRQoL: Oral health-related quality of life
PPS: Probability proportional to size
CPI: Community periodontal index
SE: Standard error
OR: Odds ratios
CI: Confidence intervals
DALYs: Disability adjusted life-years

## References

[1] World Health Organization. [https://www.who.int/health-topics/oral-health/#tab=tab_1] Accessed 5 Apr 2021.

[2] Petersen PE, Ogawa H (2018). Promoting oral health and quality of life of older people—the need for public health action. Oral Health Prev Dent.

[3] Sarita PTN, Witter DJ, Kreulen CM (2003). Chewing ability of subjects with shortened dental arches. Community Dent Oral Epidemiol.

[4] Haag DG, Peres KG, Balasubramanian M (2017). Oral conditions and health-related quality of life: a systematic review. J Dent Res.

[5] Tan H, Peres KG, Peres MA (2016). Retention of teeth and oral health-related quality of life. J Dent Res.

[6] Singh A, Gupta A, Peres MA (2016). Association between tooth loss and hypertension among a primarily rural middle aged and older Indian adult population. J Public Health Dent.

[7] Kossioni A (2018). The association of poor oral health parameters with malnutrition in older adults: a review considering the potential implications for cognitive impairment. Nutrients.

[8] Liccardo D, Cannavo A, Spagnuolo G (2019). Periodontal disease: a risk factor for diabetes and cardiovascular disease. Int J Mol Sci.

[9] Pietropaoli D, Del Pinto R, Ferri C (2018). Poor oral health and blood pressure control among US hypertensive adults: results from the national health and nutrition examination survey 2009 to 2014. Hypertension.

[10] Takeuchi K, Ohara T, Furuta M (2017). Tooth loss and risk of dementia in the community: the Hisayama study. J Am Geriatr Soc.

[11] Lee HJ, Choi EK, Park JB (2019). Tooth loss predicts myocardial infarction, heart failure, stroke, and death. J Dent Res.

[12] Feng XP (2020). Oral preventive medicine.

[13] Griffin SO, Jones JA, Brunson D (2012). Burden of oral disease among older adults and imply cations for public health priorities. Am J Public Health.

[14] Gorsuch MM, Sanders SG, Wu B (2014). Tooth loss in Appalachia and the Mississippi delta relative to other regions in the United States, 1999–2010. Am J Public Health.

[15] Wu B, Hybels CF, Liang J (2015). Social stratification and tooth loss among middle-aged and older Americans from 1988–2004. Community Dent Oral Epidemiol.

[16] Luo H, Pan W, Sloan F (2015). Forty-year trends in tooth loss among american adults with and without diabetes mellitus: an age-period-cohort analysis. Prev Chronic Dis.

[17] Li J, Xu H, Pan W, Wu B (2017). Association between tooth loss and cognitive decline: a 13-year longitudinal study of Chinese older adults. PLoS ONE.

[18] Mestaghanmi H, Labriji A, M'Touguy I (2018). Impact of eating habits and lifestyle on the oral health status of a Casablanca's academic population. Open Access Libr J.

[19] Sun HY, Jiang H, Du MQ (2018). The prevalence and associated factors of periodontal disease among 35 to 44-year-old Chinese adults in the 4th national oral health survey. Chin J Dent Res.

[20] Guo J, Ban JH, Li G (2018). Status of tooth loss and denture restoration in Chinese adult population: findings from the 4th national oral health survey. Chin J Dent Res.

[21] Marcenes W, Kassebaum NJ, Bernabé E (2013). Global burden of oral conditions in 1990–2010: a systematic analysis. J Dent Res.

[22] Wang X (2018). Report of the fourth national oral health survey in China.

[23] Yuan JQ, Lv YB, Kraus VB (2020). Number of natural teeth, denture use and mortality in Chinese elderly: a population-based prospective cohort study. BMC Oral Health.

[24] World Health Organization (WHO) (2013). Oral health surveys: basic methods.

[25] Moriya S, Ando Y, Miura H (2011). Trends and prospects of oral health conditions among Japanese: the achievement of 8020. J Natl Inst Public Health.

[26] Parker ML, Thornton-Evans G, Wei L, Griffin SO (2020). Prevalence of and changes in tooth loss among adults aged ≥50 years with selected chronic conditions—United States, 1999–2004 and 2011–2016. MMWR Morb Mortal Wkly Rep.

[27] Avenetti D, Lee HH, Pugach O, Rosales G, Sandoval A, Martin M (2020). Tooth brushing behaviors and fluoridated toothpaste use among children younger than three years old in Chicago. J Dent Child (Chic).

[28] Xu W, Lu HX, Li CR (2014). Dental caries status and risk indicators of dental caries among middle-aged adults in Shanghai. China J Dent Sci.

[29] Wang CX, Bao HL, Shen T (2013). The status and distributions of three preventive oral health behaviors among adults in China. Chin J Health Educ.

[30] Azodo CC, Umoh A (2020). Dental caries, missing teeth, and oral health behavior among smokers. Niger J Clin Res.

[31] Qu X, Qi X, Wu B (2020). Disparities in dental service utilization among adults in Chinese megacities: Do health insurance and city of residence matter?. Int J Environ Res Public Health.

[32] Naimi-Akbar A, Kjellström B, Rydén L (2019). Attitudes and lifestyle factors in relation to oral health and dental care in Sweden: a cross-sectional study. Acta Odontol Scand.

[33] Li C, Yao NA (2021). Socio-economic disparities in dental health and dental care utilisation among older Chinese. Int Dent J.

[34] Junior MFS, Sousa ACCD, Batista MJ (2017). Oral health condition and reasons for tooth extraction among an adult population (20–64 years old). Cien Saude Colet.

[35] Murakami K, Hashimoto H (2016). Wealth-related versus income-related inequalities in dental care use under universal public coverage: a panel data analysis of the Japanese study of aging and retirement. BMC Public Health.

[36] China planning development and Information Technology Department. 2018 statistic-al bulletin of China’s health development [EB/OL]. [2019–05–22]. http://www.nhc.gov.cn/guihuaxxs/s10748/201905/9b8d52727cf346049de8acce25ffcbd0.shtml

[37] Sabharwal A, Gomes-Filho IS, Stellrecht E, Scannapieco FA (2018). Role of periodontal therapy in management of common complex systemic diseases and conditions: an update. Periodontol 2000.

[38] National health Commission of the people's Republic of China. Healthy oral actionplan (2019–2025) [EB/OL]. [2019–02–16]. http://www.gov.cn/xinwen/2019-02/16/content_5366239.htm

[39] Nasseh K, Vujicic M (2017). The impact of the affordable care act's Medicaid expansion on dental care use through 2016. J Public Health Dent.

